# Cell-type annotation with accurate unseen cell-type identification using multiple references

**DOI:** 10.1371/journal.pcbi.1011261

**Published:** 2023-06-28

**Authors:** Yi-Xuan Xiong, Meng-Guo Wang, Luonan Chen, Xiao-Fei Zhang

**Affiliations:** 1 School of Mathematics and Statistics, Central China Normal University, Wuhan, China; 2 Key Laboratory of Nonlinear Analysis & Applications (Ministry of Education), Central China Normal University, Wuhan, China; 3 State Key Laboratory of Cell Biology, Shanghai Institute of Biochemistry and Cell Biology, Center for Excellence in Molecular Cell Science, Chinese Academy of Sciences, Shanghai, China; 4 School of Life Science and Technology, ShanghaiTech University, Shanghai, China; 5 Key Laboratory of Systems Health Science of Zhejiang Province, Hangzhou Institute for Advanced Study, University of Chinese Academy of Sciences, Chinese Academy of Sciences, Hangzhou, China; 6 Guangdong Institute of Intelligence Science and Technology, Hengqin, Zhuhai, Guangdong, China; Academy of Mathematics and Systems Science, Chinese Academy of Science, CHINA

## Abstract

The recent advances in single-cell RNA sequencing (scRNA-seq) techniques have stimulated efforts to identify and characterize the cellular composition of complex tissues. With the advent of various sequencing techniques, automated cell-type annotation using a well-annotated scRNA-seq reference becomes popular. But it relies on the diversity of cell types in the reference, which may not capture all the cell types present in the query data of interest. There are generally unseen cell types in the query data of interest because most data atlases are obtained for different purposes and techniques. Identifying previously unseen cell types is essential for improving annotation accuracy and uncovering novel biological discoveries. To address this challenge, we propose mtANN (**m**ul**t**iple-reference-based scRNA-seq data **ann**otation), a new method to automatically annotate query data while accurately identifying unseen cell types with the aid of multiple references. Key innovations of mtANN include the integration of deep learning and ensemble learning to improve prediction accuracy, and the introduction of a new metric that considers three complementary aspects to distinguish between unseen cell types and shared cell types. Additionally, we provide a data-driven method to adaptively select a threshold for identifying previously unseen cell types. We demonstrate the advantages of mtANN over state-of-the-art methods for unseen cell-type identification and cell-type annotation on two benchmark dataset collections, as well as its predictive power on a collection of COVID-19 datasets. The source code and tutorial are available at https://github.com/Zhangxf-ccnu/mtANN.

## Author summary

Single-cell transcriptomics is rapidly advancing our understanding of complex tissues and organisms by revealing the cellular composition of these structures. Supervised cell-type annotation is a popular approach in this field, which utilizes well-annotated single-cell RNA sequencing (scRNA-seq) references. Compared with unsupervised methods, this method saves time and effort, but it often relies on the assumption that the reference dataset includes all cell types present in the query data. Misleading predictions can result if the query dataset includes new cell types not present in the reference dataset. However, due to the diverse purposes and interests of different sequencing experiments, there are often unseen cell types in the query data that need to be identified for accurate annotation and to discover novel biological phenomena. To address this challenge, we propose mtANN, a novel method that automatically annotates query data while accurately identifying unseen cell types with the aid of multiple references. We demonstrate the annotation performance of mtANN in PBMC and Pancreas collections, where different proportions of unseen cell types are present in the query dataset. Additionally, we verify the practical application of mtANN in a collection of COVID-19 datasets for patients with different symptoms.

This is a *PLOS Computational Biology* Methods paper.

## Introduction

Single-cell RNA sequencing (scRNA-seq) technologies allow measuring the gene expression profile of individual cells, enabling the identification and characterization of the cellular composition of tissues at a previously unattainable level of resolution. Recent advances in scRNA-seq technologies have revolutionized our understanding of the heterogeneity of complex tissues. Various sequencing technologies, such as 10x Genomics Chromium, Drop-seq, and Smart-seq2, have emerged, making cell-type annotation a crucial task for analyzing new sequencing data in the context of complex tissues [1–3].

There are two typical solutions for cell-type annotation tasks. One solution is to unsupervised cluster cells into groups based on the similarity of their gene expression profiles, and annotate cell populations by assigning labels to each cluster according to cluster-specific marker genes [4–8]. However, such methods require extensive literature review and manual testing of various combinations of marker genes, which is not only time-consuming but also not reproducible across different experiments within and across research groups [9, 10]. Another solution is to learn the intrinsic relationship between gene expression profiles and cell types based on a well-annotated reference atlas, and transfer the learned relationship to query data for cell-type annotation. There are two main types of approaches to this reference-based strategy, one is to learn the similarity between the reference atlas and the query data based on statistical metrics as the basis for cell-type label transfer [11–14]. The other is to model a classifier on the reference atlas, which can make predictions directly on the query data [15–19]. The reference-based method can avoid manual selection of marker genes, and the trained classifier can be used for any new query data, providing convenience for practical applications.

Previous reference-based methods have rarely taken into account the following two issues. The first issue is the selection of the reference atlas. Noise from the reference data and incorrectly annotated cell types may lead to inaccurate annotations on the query data, and the selection of input features of the classification model can also impact the annotation performance of different methods [20, 21]. This issue can be partially addressed by integrating multiple well-annotated reference datasets and multiple gene selection methods [22–25], but an appropriate integration strategy is needed. Previous methods often integrate multiple well-labeled datasets to create a comprehensive reference atlas, which is then used to annotate the cell types in new data. However, this approach can be vulnerable to batch effects, and it is challenging to select an appropriate batch correction method in advance [26, 27]. Over-correction can lead to loss of differences between cell types in the reference data, resulting in reduced accuracy for subsequent annotations, while under-correction may not effectively address the batch effects between the datasets, increasing time and labor cost. The second issue is the difference in the joint distribution of gene expression and cell type between the reference and query datasets due to the difference in the marginal distributions. Distributional differences in gene expression, known as batch effects, have been extensively addressed in previous studies [28, 29], while differences in the distribution of cell types have been rarely considered. Discrepancies in cell types indicate that there may be cell types in the query data that are not present in the reference atlas, which can be called “unseen” cell types. Unseen cell types may suggest new biological discoveries that cannot be neglected. Additionally, ignoring the presence of unseen cell types biases the classifier learned on the reference atlas to known cell types, resulting in false predictions on the query data. These two issues are potentially related. Integrating multiple reference datasets can enrich the cell type information of reference data, but how to integrate reference datasets containing different cell types is difficult. In addition, effective methods are needed to identify cell types in the query data that are not seen in the reference data.

In order to address the above two issues, we propose mtANN (**m**ul**t**iple-reference-based scRNA-seq data **ann**otation), a novel method that automatically identifies unseen cell types while accurately annotating query dataset by integrating multiple well-annotated scRNA-seq datasets as references. The main idea of mtANN is first to learn multiple deep classification models from multiple reference datasets to obtain multiple prediction results. These results are then used to vote on metaphase annotations and to compute metrics from three complementary aspects to identify unseen cell types. Final annotations are made based on metaphase annotation and unseen cell-type identification results. mtANN has the following characteristics: (i) it utilizes the diversity of multiple reference datasets and avoids the selection of a single reference dataset; (ii) it combines the ideas of deep learning and ensemble learning to improve prediction accuracy; (iii) it proposes a new metric from three complementary aspects to measure whether a cell belongs to an unseen cell-type; and (iv) it introduces a new data-driven approach to automatically determine thresholds for the identification of unseen cell types. We benchmark mtANN using two collections of benchmark datasets, each from different tissues, sequencing technologies, and containing different cell types. We prepared a total of 75 benchmark tests, including annotations across different technologies and identification of unseen cell types belonging to different cell types. We also use a COVID-19 dataset and prepare a total of 249 tests to assess the performance. Experimental results demonstrate that mtANN outperforms state-of-the-art methods in both unseen cell-type identification and cell-type annotation.

## Results

### Overview of mtANN

**Fig 1:**
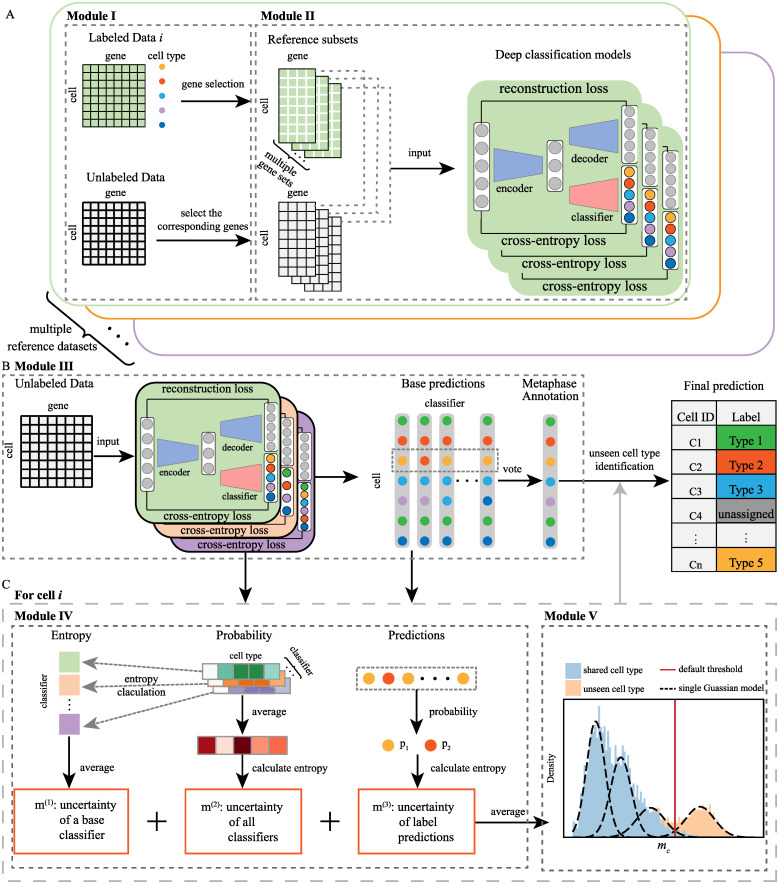
Overview of mtANN. (A) The training process of mtANN includes two modules: gene selection (Module I) and deep classification model training (Module II). The labeled data *i* is used as an example. In Module I, eight gene selection methods are applied on data *i*, obtaining multiple reference subsets. The gene sets selected by the eight gene selection methods intersect with all the genes in the query dataset, determining the input genes of multiple deep classification models. In Module II, pairs of reference subset and query dataset after gene selection are used as input to train each deep classification model. We conduct theses two modules for every labeled data, thus obtaining multiple deep classification models. (B) The prediction process of mtANN (Module III) first makes predictions for the query data based on deep classification models learned by Module II and then conducts a majority vote to obtain a metaphase annotation. (C) Unseen cell-type identification process consists of two modules: quantifying the likelihood of a cell belonging to an unseen cell type (Module IV) and using a data-driven threshold determination method to identify unseen cell types (Module V). In Module IV, we define an unseen cell-type identification metric by averaging three uncertainty measures calculated from the results obtained from III. In Module V, we derive a new a data-driven method based on Gaussian mixture model to determine the threshold for unseen type identification. If a cell is identified as belonging to an unseen cell type, mtANN annotates it as “unassigned”; otherwise mtANN annotates it as the result of module III.

The workflow of mtANN is illustrated in Fig 1 and S1 Text. mtANN consists of a training process and a prediction process, which can be divided into 5 modules to simultaneously annotate the query data and identify unseen cell types. In the training process (Fig 1A), mtANN first adopts eight gene selection methods to generate a series of subsets that retain distinct genes for each reference dataset (Module I). This step facilitates the detection of biologically important genes and increases data diversity for effective ensemble learning. Based on all reference subsets, mtANN trains a series of neural network-based deep classification models in Module II. These base classification models characterize different relationships between gene expression and cell types which are complementary in identifying unseen cell types. The prediction process contains the integration of the outputs of all base classification models and the identification of unseen cell types (Fig 1B). In Module III, mtANN obtains a metaphase annotation for query dataset by majority voting on all base results. An essential step, the identification of unseen cell types (Fig 1C) consists of two modules: the formulation of a metric for unseen cell-type identification (Module IV) and the determination of a threshold (Module V). mtANN defines a new uncertainty metric from intra-model, inter-model, and inter-prediction perspectives to identify cells that may belong to unseen cell types. Specifically, the intra-model metric quantifies uncertainty based on the average of entropy of prediction probability of different classifiers. The inter-model metric characterizes uncertainty by averaging the prediction probabilities of all classification models and then calculating the entropy. The inter-prediction metric characterizes uncertainty in terms of inconsistency among the predictions obtained by all the base classification models. Finally, based on the assumption that metric proposed in Module IV follows a mixed Gaussian distribution when there are unseen cell types in the data, mtANN fit a Gaussian mixture model to the metric to select cells with high predictive uncertainty as “unassigned” in Module V (for details please refer to Materials and method).

### Validating the effectiveness of ensemble learning in mtANN

mtANN integrates multiple well-annotated scRNA-seq datasets as references and applies eight gene selection methods to select informative genes. To validate the effectiveness of integrating multiple reference datasets and gene selection methods, we use two collections of datasets from two tissues: peripheral blood mononuclear cells (PBMC) collection which contains seven datasets sequenced by seven different technologies [20] and Pancreas collection containing four datasets sequenced by four different technologies [30–33] (Methods Datasets section). In each collection, we select one dataset as a query dataset and the rest as reference datasets alternately. We apply the eight gene selection methods, denoted as DE, DV, DD, DP, BI, GC, Disp, and Vst (see Methods Gene selection section), to these reference datasets separately, obtaining multiple reference subsets. We compare the base classification models trained on a single reference subset with mtANN, which integrates the results from different models, to demonstrate the effectiveness of ensemble learning.

**Fig 2:**
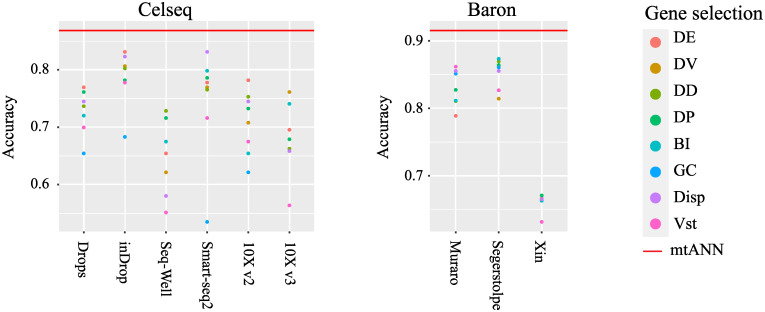
Accuracy comparison between mtANN and each base classification model. The “Celseq” dataset from PBMC collection and the “Baron” dataset from Pancreas collection, used as query datasets, are shown. In each plot, each column represents a reference dataset. Each point represents the performance of a base classification model, and points of different colors indicate different gene selection methods. The red line indicates the performance of mtANN, which integrates different reference datasets and gene selection methods.

As an illustrative example, we use “Celseq” from PBMC collection and “Baron” from Pancreas collection as the query datasets. For the PBMC collection, the remaining datasets, including “Drops”, “inDrop”, “Seq-Well”, “Smart-seq2”, “10X v2”, and “10X v3”, are all used as reference datasets. In the Pancreas collection, the “Muraro”, “Segerstolpe”, and “Xin” are used as reference datasets while “Baron” is used as the query dataset. To show the differnece in gene selection methods, we present the performance of different gene selection methods with different colored points. As shown in Fig 2, the red line is consistently higher than all the points, indicating that mtANN’s strategy of integrating all reference datasets and gene selection methods is superior to using a single classification model. It is worth noting that the performance of different gene selection methods varies across reference datasets, and no single gene selection method always outperforms others on all datasets. Similar results are also observed in experiments with other datasets from both data collections (S1 Fig). These results demonstrate the necessity and effectiveness of integrating multiple reference datasets and gene selection methods to annotate cell types in scRNA-seq datasets, highlighting the importance of leveraging diverse sources of information for accurate cell-type annotation.

### Benchmarking mtANN for unseen cell-type identification

mtANN is specifically designed for unseen cell-type identification during cell-type annotation. To demonstrate its ability in identifying unseen cell types, we also use the two data collections: PBMC and Pancreas. Within each data collection, each dataset is alternately used as a query dataset and the rest as reference datasets. To simulate an unseen cell type in the query dataset, we perform a leave-one-cell-type-out setting in each references-query pair. In doing so, we obtain a total of 50 tests in the PBMC collection and 25 tests in the Pancreas collection (for details, please refer to S2 Fig and S1 and S2 Tables). We compare mtANN with several existing popular methods, including scmap-clust, scmap-cell [11], Seurat v3 [12], ItClust [15], scGCN (entropy), scGCN (enrichment) [16], and scANVI [17] (S1 Text Methods for benchmark section) as they also provide metrics for unseen cell-type identification. We evaluate each method’s ability to distinguish unseen cell types from shared cell types by comparing their performance in terms of AUPRC scores. (S1 Text Performance assessment section).

**Fig 3:**
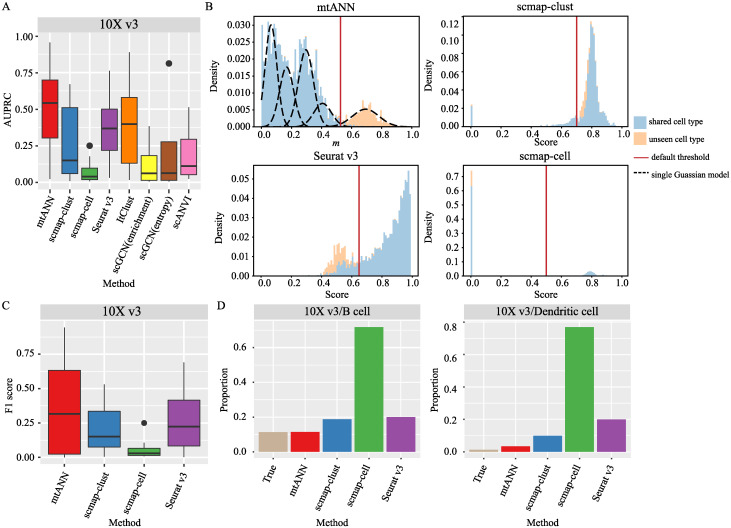
Performances in unseen cell-type identification. (A) Boxplots display the AUPRC score of mtANN and other methods using “10X v3” in PBMC collection as the query dataset. (B) Distribution of cell prediction uncertainty metrics of mtANN, scmap-clust, scmap-cell, and Seurat v3 when “10X v3” in the PBMC collection is the query dataset and “B cell” is the real unseen cell type. The histogram color distinguishes between unseen cell types and shared cell types. The black dotted line represents the subpopulations of the Gaussian mixture model fitted by mtANN, and the red solid line represents the default threshold selected by each method. Cells with metrics above the threshold are identified as “unassigned” in mtANN, while cells with scores below the threshold are identified as “unassigned” in scmap-clust, scmap-cell, and Seurat v3. (C) Boxplots display the F1 score of mtANN and other methods using “10X v3” in PBMC collection as the query dataset. The default threshold provided by each method is used to select “unassigned” cells. (D) Barplots show the proportion of unseen cell types and “unassigned” cells predicted by each method using the “10X v3” dataset in the PBMC collection as the query dataset. The real unseen cell type is indicated in the title of each plot.

The results presented in Fig 3A show that mtANN outperforms the compared methods when the “10X v3” dataset is the query. The results on other datasets also demonstrate the superior performance of mtANN (S3 Fig). Across all the experiments, we count the number of times each method ranks first in terms of AUPRC scores. We observe that the performance of scmap-clust, ItClust, and scGCN (enrichment) vary widely between different data collections (S4 Fig). These methods may rank first in some datasets but have a large performance drop in others, possibly due to differences in the distribution of cell types in the query and reference datasets. Take ItClust as an example, missing cell types in the reference data may lead to misalignment of cell labels and clusters, resulting in over-fitting of the model. mtANN effectively addresses this issue by borrowing complementary information between different reference datasets to define the uncertainty of cell annotation at different aspects, thereby accurately distinguishing between shared cell types and unseen cell types (Fig 3B and S5–S7 Figs).

Another important issue in identifying unseen cell types is the choice of thresholds. Most methods for identifying unseen cell types use a fixed threshold (e.g., scmap) or a fixed ratio (e.g., Seurat v3) as the threshold, which may not generalize well on new datasets. To test the performance of the threshold selection methods, mtANN is compared with scmap-clust, scmap-cell and Seurat v3, which have provided threshold selection methods, in terms of F1 score. The results are presented in Fig 3C and S8 Fig. It can be seen that mtANN performs better than other methods in most cases, scmap-cell performs the worst in PBMC collection, and the relative performance of scmap-clust, scmap-cell and Seurat v3 varies by dataset. To further investigate the reasons for the differences in the performance of these methods, we compare the proportion of true unseen cell types with the proportion of cells identified as “unassigned” by each method (S9 Fig). As an example, we take an experiment where “10X v3” is the query dataset (Fig 3D). When the unseen cell type is B cell, the true proportion of the unseen cell type is 11%, and the proportion of cells predicted by mtANN as “unassigned” is close to 11%. However, the proportion of “unassigned” cell predicted by scmap-cell is much higher than the true proportion, and the proportion of “unassigned” cells identified by Seurat v3, fixed at 20%, is also higher than the true proportion. When the unseen cell type is Dendritic cell, the proportion of unseen cell types is small. The proportion of cells predicted by mtANN as “unassigned” decreases and is close to the true proportion, while the proportions of cells predicted by scmap-clust, scmap-cell, and Seurat v3 as “unassigned” are much higher than the true proportion. With default thresholds, we count the number of times each method ranks first in terms of F1 score across all the experiments, and find that mtANN is consistently able to accurately identify unseen cell types when the proportion of unseen cell types is varied (S4 Fig).

### Benchmarking mtANN for cell-type annotation

In addition to identifying unseen cell types, annotating new query data requires labeling cells belonging to shared types. To evaluate the performance of mtANN in annotating query datasets with unseen cell types, we also use the PBMC and Pancreas collections to conduct the experiments. In each experiment, one dataset is selected as the query dataset, while the remaining ones are used as reference datasets. To account for the presence of unseen cell types, we still use the leave-one-cell-type-out setting in each experiment. As the choice of threshold can affect the annotation accuracy of the query dataset, we evaluate the performance with two different approaches for threshold selection: using the real proportion of unseen cells and using the default threshold provided by each method.

**Fig 4:**
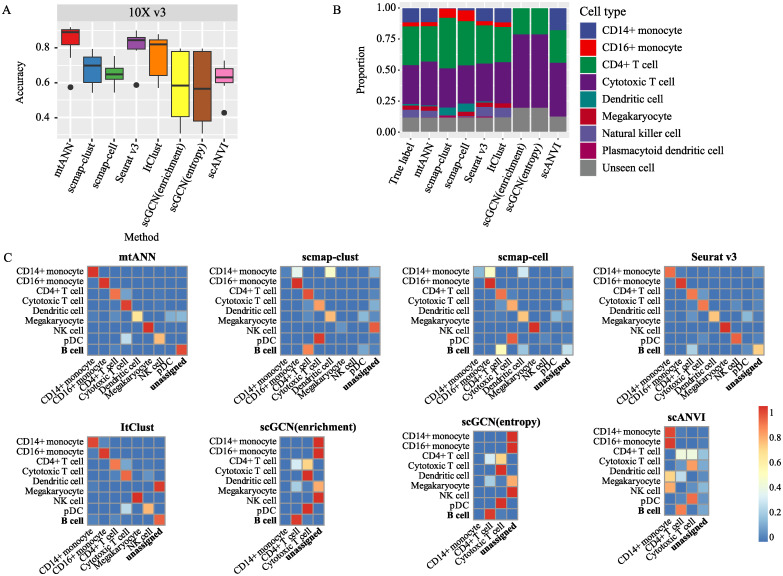
Performances in cell-type annotation when there are unseen cell types in query dataset. (A) Boxplots show the accuracy of mtANN and other methods when the query dataset is “10X v3” dataset in the PBMC collection. (B) Bar plots display the real proportion of cell types and the proportion of cell types annotated by each method when the query dataset is “10X v3” dataset in the PBMC collection, with the real unseen cell type being “B cell”. (C) Heatmaps depict the confusion matrices of mtANN and other methods when the query dataset is the “10X v3” dataset and the real unseen cell type is “B cell”. The confusion matrix shows the proportion of cells belonging to one cell type that are predicted to be of other cell types, with the row and column names corresponding to the true cell labels and the predicted cell labels of the query dataset, respectively. The abbreviations NK cell and pDC refer to the Natural killer cell and the Plasmacytoid dendritic cell, respectively.

When using the actual proportion (let *p*) of unseen cell types in the query dataset to determine threshold, we calculate the threshold as the value corresponding to the ((1 − *p*) * 100)% quantile of the metrics of mtANN and scGCN (entropy) and the (*p* * 100)% quantile of the metrics of other methods. The annotation accuracy of mtANN and other methods are presented in Fig 4A and S10 Fig. It can be observed that in different experiments with varying proportions of unseen cell types, mtANN consistently achieves higher annotation accuracy than other methods (S4 Fig). The performances of scmap, Seurat v3, and ItClust vary greatly across different experiments. This may be attributed to the presence of unseen cell types in the query dataset, resulting in annotation bias towards shared cell types. To validate this, we calculate the Pearson correlation coefficient between the true proportional distribution of cell types in each experiment and the proportional distribution of the predicted results of each method (S11 Fig). The results show that mtANN has the highest correlation in both PBMC and Pancreas collections. We also use “10X v3” datasets as the query dataset and B cell as the real unseen cell type to provide an example. Fig 4B shows that the proportion of cell types obtained from mtANN’s prediction is more similar to the true proportion. In detail, we find that mtANN identifies most B cells as “unassigned”, whereas all other comparison methods annotate most B cells as a similar cell type (CD4+ T cells) as they are all derived from lymphoid progenitors (Fig 4C). For shared cell types, mtANN performs better at distinguishing the two monocyte subtypes, while scmap-clust and scmap-cell tend to confuse CD16+ monocyte cells with CD14+ monocyte cells. scGCN (enrichment), scGCN (entropy), and scANVI fail to annotate monocytes and other rare cell types (Dendritic cell, Megakaryocyte, Natural killer cell, and Plasmacytoid dendritic cell). As ItClust and scGCN are not designed for multiple reference datasets, we also use combat [34] to correct batch effects between different reference datasets before combining them, and compare the annotation results of the corrected reference data and the directly combined reference data (S1 Text Methods for benchmark section). For most datasets, the corrected reference datasets perform worse than the directly combined reference datasets (S12 Fig). This may be partially due to the fact that even though batch effect correction can remove batch effects between different reference datasets to some degree, over-correction may occur, and the distribution diversity of the reference dataset, which may be important for annotating query data, decreases.

In reality, obtaining the real proportion of unseen cell types is often not feasible, making the default threshold provided by each method more practical and essential. The prediction accuracies of mtANN, scmap-clust, scmap-cell, and Seurat v3 when using the default method to select the threshold are presented in S13 Fig. We can observe that the accuracy of mtANN is higher than those of the compared methods when “Celseq”, “Drops”, “inDrop”, “Smart-seq2”, “10X v2”, and “10X v3” are evaluated as the query datasets (S13(A) Fig). Furthermore, S13(B) Fig shows that mtANN also has the best performance when “Baron” and “Xin” are used as the query datasets. In addition, the result of mtANN at the default threshold is similar to the result at the actual proportion (S14 Fig), indicating that the threshold selected by mtANN is comparable to the threshold determined according to actual proportion of unseen cells.

### Effect of number of reference datasets on performance

**Fig 5:**
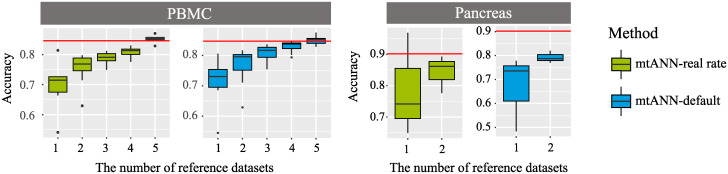
Accuracy comparison of mtANN with varying numbers of references. The query datasets used are the “10X v2” dataset from the PBMC collection and the “Baron” dataset from the Pancreas collection. Each plot displays the accuracy on the *y*-axis and the number of references used in mtANN annotation on the *x*-axis. The red line in each plot represents the performance of mtANN when integrating all the remaining data as reference datasets. Results are presented using both the threshold determined by the actual proportion of unseen cells and the default threshold provided by mtANN.

In this section, we conduct experiments with PBMC and Pancreas collections to evaluate the effect of the number of reference datasets on mtANN annotation results. We use “10X v2” in the PBMC collection and “Baron” in the Pancreas collection as the query datasets, and B cell and acinar cell are used as unseen cell types, respectively. We run mtANN with a subset of the remaining datasets with different numbers of datasets. For the PBMC collection, we try all possible combinations of 1, 2, 3, 4, 5, and 6 datasets, resulting in 6, 15, 20, 15, 6, and 1 results, respectively. For the Pancreas collection, we run mtANN with all possible combinations of 1, 2, and 3 datasets, resulting in 3, 3, and 1 results. As shown in Fig 5, the annotation accuracy shows an upward trend with the increase in the number of reference datasets in both the PBMC and Pancreas collections. Additionally, in the PBMC collection, the combination of some 5 datasets in the remaining 6 datasets can achieve satisfactory results, but it is difficult to know which data should be selected in practical applications. Thus, multiple reference datasets are helpful for improving annotation performance.

### Ablation study on metrics for unseen cell-type identification

**Fig 6:**
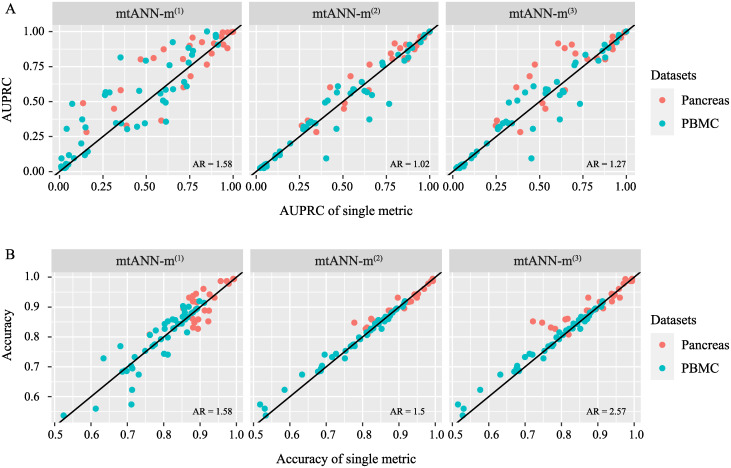
Comparisons between the combination of the three metrics for determining unseen cell types and each individual metric. (A) The comparison in unseen cell-type identification. (B) The comparison in cell-type annotation. Each dot in the plot represents an experiment, and the different colors of the dots represent the data collections. The *x*-axis represents the AUPRC (panel A) or accuracy (panel B) of a single metric, and the *y*-axis represents the AUPRC (panel A) or accuracy (panel B) of mtANN. The black solid line represents the line of *y* = *x*, indicating the situation where mtANN and the single metric have the same performance. The AR index is reported to quantify the performance comparison, with a value greater than 1 indicating that mtANN outperforms the corresponding single metric.

We further investigate whether combining the three complementary measurements of uncertainty provides superior performance compared to using a single evaluation metric. We run mtANN with four different settings: using only one of the three metrics *m*^(1)^, *m*^(2)^, and *m*^(3)^, and using a combination of the three metrics (*m*) for determining unseen cell types. We evaluate unseen cell-type identification accuracy using AUPRC and cell type annotation performance in terms of accuracy. To facilitate comparison, we introduce the Accuracy Ratio (AR) index (S1 Text Performance assessment section), which represents the ratio of the number of tests in which one setting outperforms another setting to the number of tests in which it performs worse. An AR greater than 1 indicates that the former setting performs better than the latter. We present the comparison results in Fig 6. Our findings indicate that the ensemble uncertainty measurement in unseen cell-type identification (Fig 6A) and cell-type annotation (Fig 6B) outperforms the three single metrics. Results shown in S15 Fig demonstrate that each single metric has its own advantages and limitations and none of them can perform well in all experiments. For example, while *m*^(2)^ generally outperforms *m*^(1)^ in most cases, there are some instances where the accuracy of *m*^(1)^ surpasses that of *m*^(2)^. Therefore, we conclude that a more comprehensive uncertainty measurement scheme that combines complementary metrics provides better performance compared to individual metrics.

### Cell-type annotation of COVID-19 patients with different symptoms

Coronavirus disease 2019 (COVID-19) has caused more than 647 million infections and more than 6.6 million deaths, according to World Health Organization (WHO) statistics as of December 16, 2022. It is thus important to annotate the cell types of the sequencing data from COVID-19 patients for understanding the disease mechanism. With many scRNA-seq data from COVID-19 patients available, we select the study of COVID-19 that offers a comprehensive immune landscape [35], including 284 samples from 196 COVID-19 patients and controls to assess the performance of mtANN on real data. We use the dataset from PBMC cells in the COVID-19 dataset as the query datasets and the PBMC collection we used above [20] as references to evaluate the performance of mtANN and other methods.

**Fig 7:**
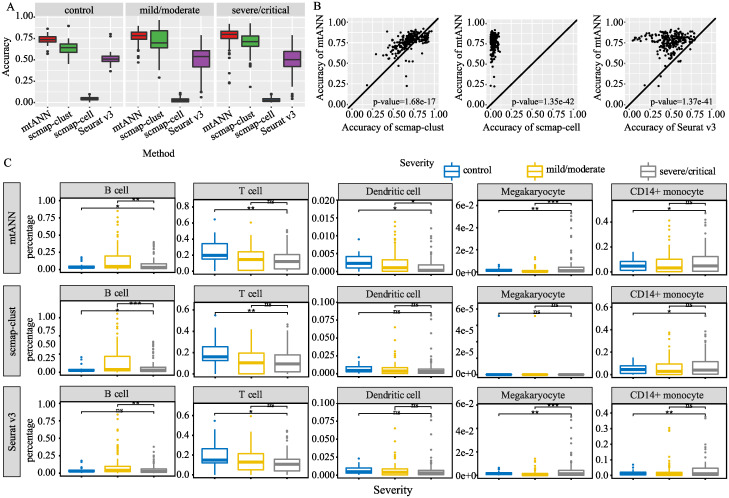
Application of different methods on COVID-19 dataset. (A) Boxplots display the accuracy of different methods on samples with different symptoms. (B) One-to-one comparisons are made between mtANN and other methods including scmap-clust, scmap-cell, and Seurat v3. Each point in the plot represents a query dataset. The *p*-values of two-sided paired Wilcoxon signed-rank tests are reported to test the significance of differences in performance. (C) Boxplots show the proportions of B cells, T cells, Dendritic cells, Megakaryocyte cells, and CD14+ monocytes between samples with different symptoms. The significance of the two-sided T-test is represented by stars, where one, two, and three stars indicate p-values less than 0.05, 0.01, and 0.001, respectively. The abbreviation “ns” means that the corresponding p-value is greater than 0.05.

We group the cells according to samples’ id, resulting in 249 query datasets. mtANN is compared with scmap-clust, scmap-cell, and Seurat v3 under the default threshold parameters for identifying unseen cell types. The accuracies of mtANN and other methods on the 249 query datasets are presented in Fig 7A. It can be seen that the accuracies of mtANN for patients with different symptoms are higher than other methods, and scmap-cell suffers a decrease. We further conduct a one-to-one comparison and find that mtANN significantly (two-sided paired Wilcoxon test, p-value < 0.01) outperforms the compared methods (Fig 7B). We compare the composition of cell types between patients with different symptoms and find that the proportion of B cells increases in patients with severe symptoms, and the percentage of Dendritic cells and T cells decreases, particularly in patients with severe symptoms (Fig 7C), which is consistent with the lymphopenia phenomenon previously reported [36]. We also find that the percentage of Megakaryocyte and CD14+ monocyte elevates in patients with severe symptoms, which is agreement with the original study [35]. Compared with scmap-clust and Seurat v3, mtANN can more accurately reflect the difference in the proportion of Dendritic cell and Megakaryocyte cells between different populations, which is instructive for the study of the development process of COVID-19.

## Discussion

With the development of single-cell sequencing technology, traditional unsupervised clustering-based cell-type annotation methods are difficult to adapt to rapidly generated datasets since they are time-consuming [37, 38]. Another method for automatic cell-type annotation based on a reference atlas has been widely studied, but these methods are rarely able to discover unseen cell types in the query data [23, 39, 40]. Identifying previously unseen cell types can lead to new biological discoveries, while errors in identification may result in missing new biological discoveries or leading to improper biological conclusions. Although some previous methods for automatic cell-type annotation address the problem of identifying unseen cell types, they all rely on setting a default threshold instead of proposing a methodology for automatically selecting a threshold. The choice of threshold can significantly impact the accuracy and usability of the method.

In this study, we propose a novel ensemble learning-based cell-type annotation method to automatically annotate cell-type labels for query datasets. Our method mainly has three innovations: (i) it integrates multiple reference datasets not only to enrich cell types in the reference atlas, but also to provide complementary information to annotate cell types; (ii) it proposes a new metric from three complementary aspects to effectively measure whether a cell belongs to an unseen cell type; and (iii) it proposes a data-driven approach to adaptively determine the threshold for unseen cell-type identification. Through the 75 experiments, we demonstrate the annotation ability of mtANN for new sequencing data and validate that mtANN can accurately distinguish between unseen cell types and shared cell types, even when the proportion of unseen cell types in the query dataset varies. Additionally, mtANN has excellent discrimination between two similar cell types in the shared cell types. Our application on the real data verifies the annotation performance of mtANN for COVID-19 patients and shows the difference in the proportions of different immune cells between different populations. We also compare the runtime and memory usage of mtANN with other methods (S16 Fig). Despite integrating multiple reference datasets and gene selection methods, mtANN’s runtime is still comparable, and it uses less memory. Moreover, mtANN allows users to input pre-selected gene sets or gene sets of interest, which can further reduce the running time. Our comprehensive benchmark and extensive application on publicly available benchmark datasets indicate that mtANN has achieved state-of-the-art performance for unseen cell-type identification and cell-type annotation in the meantime.

There may be two limitations in integrating multiple reference datasets for unseen cell-type identification that we have not addressed well in this work. One is the inconsistent terminology of cell types across different reference datasets. In this work, we address this problem by manually checking the cell-type annotations. For example, the cell type “PP” in the Xin dataset is changed to “gamma”, as “gamma” is the name used by all other datasets. Several approaches can be attempted in the future to match cell types between datasets, such as matching based on marker genes of cell types or mutual prediction between datasets. In this work, we only mark the cells that are considered to belong to unseen cell types as “unassigned”. Thus, another limitation is that we do not provide a further biological interpretation of these cells. A straightforward way to determine the identities of these cells is to use unsupervised annotation methods. In addition, integrating the Cell Ontology [41, 42] into the method may be instructive for annotation of “unassigned” cells. For example, when Plasmacytoid dendritic cells are absent from reference dataset, they can be assigned to supertypes of Dendritic cells with the help of Cell Ontology. In the future, we will extend our method to implement this functionality.

## Materials and methods

### Notations and problem statement

For convenience, we first introduce some notations (S3 Table). We assume that *M* well-labeled reference datasets with the same tissue type as the query dataset are collected. Let $\{(X^{r_{i}},Y^{r_{i}}){\}}_{i=1}^{M}$ denote the references, where $X^{r_{i}}$ is an $n^{r_{i}}\times p^{r_{i}}$ matrix that denotes the gene expression matrix after library size normalization of the *i*-th reference dataset with rows representing cells and columns representing genes, and $Y^{r_{i}}$ denotes the corresponding cell type labels. The number of cells and genes of the *i*-th reference dataset are denoted by $n^{r_{i}}$ and $p^{r_{i}}$ separately. Let $K^{r_{i}}$ denote the set of cell types observed in $Y^{r_{i}}$ and $K=union(\{K^{r_{i}}{\}}_{i=1}^{M})$ denotes all cell types present in all reference datasets. Let *X*^*q*^ be an *n*^*q*^ × *p*^*q*^ matrix that denotes the gene expression matrix after library size normalization of the query dataset. The number of cells and genes of the query dataset are denoted by *n*^*q*^ and *p*^*q*^ separately. Let *Y*^*q*^ denotes the corresponding cell type labels which is unknown.

In this study, we focus on annotating cells in a new query dataset with multiple well-labeled references. Mathematically, our goal is to estimate *Y*^*q*^ based on observed data, $\{(X^{r_{i}},Y^{r_{i}}){\}}_{i=1}^{M}$ and *X*^*q*^. In practical application, although multiple reference datasets are integrated, there may still be cell types in the query dataset that are not observed in any reference dataset. We call such cell types “unseen cell types”. Identifying cells belonging to unseen cell types while accurately annotating other types is essential. To achieve our goal, we propose a novel multiple-reference-based scRNA-seq data annotation method (Fig 1 and S1 Text The workflow of mtANN section). Our method consists of five modules. First, we adopt eight gene selection methods to generate a series of subsets that retain distinct genes for each reference dataset. Second, we train a series of neural network-based deep classification models based on all subsets of all reference datasets. Third, we obtain a metaphase annotation for query dataset through integrating the base results output by all base classification models. Fourth, a new metric for prediction uncertainty measurement is identified from three complementary aspects, distinguishing unseen cell types from shared cell types. Finally, we fit a Gaussian mixture model to the prediction uncertainty metric, choosing a threshold based on the grouping of cells. With Module IV and Module V, we identify cells that may belong to unseen cell types and mark them as “unassigned”.

### Module I: Gene selection

In order to include as much informative gene sets as possible with different meanings, we selecte eight gene selection methods, including five supervised gene selection methods: Limma, Bartlett’s test, Kolmogorov-Smirnov test, Chi-squared test, and Bimodality index, which are collected by scClassify [23]; and three widely used unsupervised methods for highly variable gene selection, including Gini index [43], Dispersion, and Variance-stabilizing transformation [12] (for details, please refer to S1 Text). For each reference dataset $\left(X^{r_{i}},Y^{r_{i}}\right)$, we apply the eight gene selection methods to pick genes from different perspectives which are differentially expressed genes (DE), differential variable genes (DV), differentially distributed genes (DD), differentially proportioned genes (DP), and bimodally distributed genes (BI), and the highly variable genes based on Gini index-based clustering (GC), dispersion (Disp), and variance (Vst) (the parameter settings can be found in S4 Table). We index these gene selection methods using *j* = 1, ⋯, 8. Let $G^{r_{ij}}$ denotes the gene set selected by the *j*-th gene selection method for the *i*-th reference dataset, where *r*_*ij*_, *i* = 1, ⋯, *M*, *j* = 1, ⋯, 8 is the index of reference subsets, and *G*^*q*^ denote all genes in the query dataset. By doing so, we can obtain 8*M* reference subsets to expand the diversity of references. In each reference subset, we can train a deep classification model with $G^{r_{ij}}\cap G^{q}$ as the input features. We denote $X^{r_{ij}}$ and $X^{q_{ij}}$ as the gene expression matrix after gene selection for the *ij*-th reference subset and query dataset. For convenience, we still denote the preprocessed data by $X^{r_{ij}}$ and $X^{q_{ij}}$. Based on preprocessed data, we construct a dataset pair $\left(X^{r_{ij}},Y^{r_{i}},X^{q_{ij}}\right)$ as the training dataset for the next step to train a base classification model.

### Module II: Deep classification model training

Based on each dataset pair $\left(X^{r_{ij}},Y^{r_{i}},X^{q_{ij}}\right)$, we train a classification model based on deep learning. The classification model involves two components: the embedding component for extracting cell type-related features and the linear classifier layer for classification. Let *E*^*ij*^ and *C*^*ij*^ denote the embedding component and the linear classifier layer separately. The forward propagation result of the classification model after softmax transformation can be defined as ${\hat{P}}^{r_{ij}}=softmax(C^{ij}(E^{ij}(X^{r_{ij}})))$, where ${\hat{P}}^{r_{ij}}$ is an assignment probability matrix with rows representing cells and columns representing cell types. The (*c*, *k*)-th element of ${\hat{P}}^{r_{ij}}$ can be regarded as the predicted probability of cell *c* in (*ij*)-th reference subset belonging to cell type *k*. The cross-entropy loss

$$\begin{array}{c} L_{ce}=-\frac{1}{n^{r_{i}}}\sum_{c=1}^{n^{r_{i}}}\sum_{k\in K}\;1_{\left[Y_{c}^{r_{i}}=k\right]}\;\text{log}\;{\hat{P}}_{ck}^{r_{ij}}. \end{array}$$ (1)

is used to train the classification model, where **1**_[⋅]_ denote the indicative function, and $n^{r_{i}}$ is the number of cells in *i*-th reference dataset.

To enable the embedding component *E*^*ij*^ to fully capture the characteristics of cells and make the classification model better fit the query dataset, we employ the embedding component as an encoder and use a mirror image of the embedding component as a decoder to construct an autoencoder. The reconstruction loss of cells both from the reference subset and the query subset is taken into consideration when training the classification model. Let *D*^*ij*^ denote the decoder component. The forward propagation results of the autoencoder can be defined as ${\hat{X}}^{r_{ij}}=D^{ij}(E^{ij}(X^{r_{ij}}))$ and ${\hat{X}}^{q_{ij}}=D^{ij}(E^{ij}(X^{q_{ij}}))$, where ${\hat{X}}^{r_{ij}}$ and ${\hat{X}}^{q_{ij}}$ denote the reconstruction of $X^{r_{ij}}$ and $X^{q_{ij}}$ separately. The reconstruction loss is measured by the mean squared error, which can be formulated as

$$\begin{array}{c} L_{re}=\frac{1}{n^{r_{i}}p^{ij}}\|{\hat{X}}^{r_{ij}}-X^{r_{ij}}{\|}_{F}^{2}+\frac{1}{n^{q}p^{ij}}\|{\hat{X}}^{q_{ij}}-X^{q_{ij}}{\|}_{F}^{2}, \end{array}$$ (2)

where *p*^*ij*^ represents the number of genes in this dataset pair, and ‖⋅‖_*F*_ denote the Frobenius norm of a matrix.

Therefore, the final optimization problem for training the classification model for dataset pair $\left(X^{r_{ij}},Y^{r_{i}},X^{q_{ij}}\right)$ can be written as

$$\begin{array}{c} \underset{E^{ij},D^{ij},C^{ij}}{\text{min}}\;L_{ce}+\lambda L_{re}, \end{array}$$ (3)

where λ is the tuning parameter and the default value is 1. Details of the neural network architecture, hyperparameter settings, and initialization can be found in S1 Text. For all the reference-query pairs, we can have 8*M* base classification models denoted by {(*E*^*ij*^, *C*^*ij*^)}_*i*=1,⋯,*M*,*j*=1,⋯,8_.

### Module III: Query dataset annotation

Based on one base classification model (*E*^*ij*^, *C*^*ij*^), we take the corresponding query subset $X^{q_{ij}}$ as input. The forward propagation result along the model after softmax transformation can be formulated as ${\hat{P}}^{q_{ij}}=softmax(C^{ij}(E^{ij}(X^{q_{ij}})))$. The (*c*, *k*)-th element of ${\hat{P}}^{q_{ij}}$ can be regarded as the predicted probability of cell *c* in the query dataset belonging to cell type *k*. For each cell in the query dataset, we obtain *q*_*ij*_-th base prediction label ${\hat{Y}}^{q_{ij}}$ according to ${\hat{P}}^{q_{ij}}$. For cell *c*,

$$\begin{array}{c} {\hat{Y}}_{c}^{q_{ij}}=\underset{k\in K}{\text{arg}\;\text{max}}\;{\hat{P}}_{ck}^{q_{ij}}. \end{array}$$ (4)

Then, based on the majority voting principle we integrate all these predictions for consensus annotation, denoted by ${\hat{Y}}^{q}$. For cell *c*, we calculate

$$\begin{array}{c} {\hat{Y}}_{c}^{q}=\underset{k\in K}{\text{arg}\;\text{max}}\frac{\sum_{i=1}^{M}\sum_{j=1}^{8}1_{\left[{\hat{Y}}_{c}^{q_{ij}}=k\right]}}{L_{k}}, \end{array}$$ (5)

where $L\in R^{K}$, and *L*_*k*_ indicates the number of reference subsets which contain cells belong to cell type *k*. The numerator represents the number of times that cell *c* is predicted to belong to cell type *k* across all base predictions and the denominator represents the number of reference subsets containing cell type *k*. The role of the denominator is to handle the situation where a cell is predicted as a single-reference-specific cell type and indeed belongs to that cell type in the query dataset. It is worth stating that the setting of the denominator increases the prediction probability of the single-reference-specific cell type, making full use of the diversity of the reference datasets. Details of the integration can be found in S1 Text.

### Module IV: Metrics for unseen cell identification

Since there is no training data in reference datasets for the unseen cell types, the predictions for the cells belonging to these cell types can be more uncertain. We define the uncertainty from three perspectives based on the outputs of all the base classification models, including the intra-model, inter-model, and inter-prediction perspectives. For a cell belonging to the unseen cell type, from the intra-model perspective, no single cell type dominates the predicted probabilities in all base classifiers; From the inter-model perspective, no cell type has a high prediction confidence among the overall predicted probabilities of all base classifiers; From the inter-prediction perspective, there is a large inconsistency among the predictions obtained by all the base classification models. Therefore, we design three entropy-based measures, denoted by *m*^(1)^, *m*^(2)^ and *m*^(3)^, to quantitatively characterize the uncertainty, where *m*^(1)^ is from the intra-model perspective, *m*^(2)^ is from the iner-model perspective, and *m*^(3)^ is from the inter-prediction perspective.

#### Intra-model measurement from each single classification model

The first metric *m*^(1)^ calculates the entropy of the probability that a cell belongs to different cell types by each classification model, and then averages these entropy values as a final uncertainty measure. For cell *c*, this metric is defined as

$$\begin{array}{c} m_{c}^{\left(1\right)}=\frac{1}{8M}\sum_{i,j}H({\hat{P}}_{c.}^{q_{ij}}), \end{array}$$ (6)

where *H*(⋅) represents the function to compute an entropy and is defined as $H({\hat{P}}_{c.}^{q_{ij}})=-\sum_{k\in K}{\hat{P}}_{ck}^{q_{ij}}\;{\text{log}}_{2}({\hat{P}}_{ck}^{q_{ij}})$. The larger $m_{c}^{\left(1\right)}$ is, the more uncertain the predictions is, and thus the more likely the cell *c* is of unseen cell types.

#### Inter-model measurement from the overall predicted probabilities

The second measure *m*^(2)^ characterizes uncertainty from the inter-model perspective by first averaging the prediction probabilities of all classification models and then calculating the entropy. We compute the average of prediction probabilities *Q*^(2)^ as

$$\begin{array}{c} Q_{ck}^{\left(2\right)}=\frac{\sum_{i,j}{\hat{P}}_{ck}^{q_{ij}}}{L_{k}}, \end{array}$$ (7)

where $Q_{ck}^{\left(2\right)}$ represents the average of the prediction probability that cell *c* belongs to cell type *k* across all classification models. Then, *Q*^(2)^ is transformed into a probability matrix ${\tilde{Q}}^{\left(2\right)}$ by dividing each value by the row sum. For cell *c*, if there is no cell type with high prediction confidence in ${\tilde{Q}}_{c.}^{\left(2\right)}$, then the prediction uncertainty of cell c is high. Therefore, we define *m*^(2)^ as the entropy of the average of the prediction probability. For cell *c*, it is defined as

$$\begin{array}{c} m_{c}^{\left(2\right)}=H({\tilde{Q}}_{c.}^{\left(2\right)}). \end{array}$$ (8)

The larger $m_{c}^{\left(2\right)}$ indicates the the more uncertainy, and thus cell *c* is more likely to be of an unseen cell type.

#### Inter-prediction measurement from the hard-assignment labels

The third measure *m*^(3)^ calculates uncertainty from the inter-prediction perspective. The difference with *m*^(2)^ is that it integrates the hard-assignment labels of all classification models, rather than the prediction probabilities. Let *Q*^(3)^ denotes the integration result for this measure. The (*c*, *k*)-th element of *Q*^(3)^ is defined as

$$\begin{array}{c} Q_{ck}^{\left(3\right)}=\frac{\sum_{i,j}\;1_{\left[{\hat{Y}}_{c}^{q_{ij}}=k\right]}}{L_{k}}. \end{array}$$ (9)

Then, as before, we transform *Q*^(3)^ into a probability matrix ${\tilde{Q}}^{\left(3\right)}$ by dividing each value by the row sum. If the different base prediction labels for cell *c* are inconsistent, then none of cell types dominate the row *c* of ${\tilde{Q}}^{\left(3\right)}$. Similarly, we calculate the entropy to define *m*^(3)^, i.e., for cell *c*,

$$\begin{array}{c} m_{c}^{\left(3\right)}=H({\tilde{Q}}_{c.}^{\left(3\right)}). \end{array}$$ (10)

After obtaining the three complementary metrics, *m*^(1)^, *m*^(2)^ and *m*^(3)^, the values are scaled to [0, 1] linearly through Min-Max scaling separately, denoted by ${\bar{m}}^{\left(1\right)}$, ${\bar{m}}^{\left(2\right)}$ and ${\bar{m}}^{\left(3\right)}$. The ensemble uncertainty measure *m* is defined as the average of these three measures which is

$$\begin{array}{c} m=\frac{{\bar{m}}^{\left(1\right)}+{\bar{m}}^{\left(2\right)}+{\bar{m}}^{\left(3\right)}}{3}. \end{array}$$ (11)

Generally, for cell *c*, a larger value of *m*_*c*_ indicates a higher probability that cell *c* belongs to an unseen cell type. Details of the calculation of each measurement can be found in S1 Text.

### Module V: Data-driven method for default threshold selection

Determining the threshold to distinguish cells belonging to unseen cell types remains subjective in previous studies, and a method to automatically determine the exact threshold is required. Instead of using a fixed value as a threshold as in previous studies, we provide a new method to automatically identify cells with higher uncertainty. We initially apply a Gaussian mixture model to the uncertainty metric *m*, with the number of mixture components ranging from 1 to 5. The optimal number of components is determined based on the Akaike information criterion (AIC). If the suitable number of mixture components determined by AIC is 1, we consider that no cells are assigned as “unassigned”. Otherwise, all the cells are divided into different groups according to the posterior probability of the estimated Gaussian mixture model, and then the mean of the metric *m* of cells within each group is calculated. If there are groups with a mean greater than or equal to 0.6, these groups are considered to be uncertain groups. Meanwhile, the group with the largest mean is considered to be the uncertain group. All the cells in the uncertain groups are annotated as “unassigned”.

### Datasets

We use two collections of publicly available scRNA-seq datasets and a study of COVID-19 patients (S5 and S6 Tables) varying from tissues (peripheral blood mononuclear cells (PBMC) and Pancreas), cell populations and sequencing technologies to benchmark mtANN and other methods.

The PBMC collection, including seven datasets curated from Butler et al. [44], are sequenced by Cel-seq, Drops, inDrop, Seq-Well, Smart-seq2, 10X v2, and 10X v3. The datasets are downloaded from https://doi.org/10.5281/zenodo.3357167 [20]. The Pancreas collection, including four datasets curated from Baron et al. [30], Muraro et al. [31], Segerstolpe et al. [32], and Xin et al. [33], are sequenced by inDrop, Cel-seq2, Smart-seq2, and SMARTer. We obtain all the datasets from https://hemberg-lab.github.io/scRNA.seq.datasets/human/Pancreas/. Following the study of scClassify [23], we manually check the cell-type labels that are provided by the original authors of each dataset and remove the cell types that are labeled as “unclear” in the Muraro dataset, “co-expression”, “not applicable”, “unclassified” and “unclassified endocrine” in Segerstolpe dataset, and “alpha.contaminated”, “beta.contaminated”, “delta.contaminated” and “gamma.contaminated” in Xin dataset.

The study of COVID-19 [35] provides a scRNA-seq atlas including 284 samples from PBMC, bronchoalveolar lavage fluid (BALF), sputum, and pleural fluid mononuclear cells (PFMCs) which is available at GEO database: GSE158055. In this study, we only take 249 of these samples from PBMC. We manually renamed CD8+ T cells to Cytotoxic T cells to be consistent with the previous PBMC collection.

For the PBMC and Pancreas data collections, we first remove cell types with less than 10 cells, then genes expressed in less than 100 cells are removed, and cells expressing less than 100 genes are later removed. These datasets are selected to be either the reference or the query datasets in the following experiments. For details about the reference and query datasets used in the benchmark tests, please refer to S1 and S2 Tables.

### Data preprocessing

For each scRNA-seq dataset, preprocessing consists of four steps. Firstly, the library size normalization is performed, i.e., dividing the expression of each gene in a cell by the total expression of the cell and then multiplying it by a scale factor of 10000 in order to make the total expression values of all cells after being transformed the same. Secondly, logarithmic transformation is applied to the dataset to make each expression value *x* be log_2_(*x* + 1). Thirdly, z-score standardization is performed for each gene so that the mean of each gene on all cells is equal to 0 and the standard deviation of each gene is equal to 1. Lastly, the expression values of each gene are scaled to [0, 1] linearly through Min-Max scaling. It is worth noting that the first step is applied to the raw datasets, while the last three steps are applied to the datasets after gene selection.

## Supporting information

S1 Fig Accuracy comparison between mtANN and each base classification model. Each plot is named after the corresponding query dataset. In each plot, each column represents a reference dataset, and each point represents the performance of a base classification model, with points of different colors indicating different gene selection methods. The red line indicates the performance of mtANN, which integrates different reference datasets and gene selection methods. Two collections are used in this comparison: (A) PBMC collection, and (B) Pancreas collection.
(EPS)

S2 Fig Illustration of experimental design. To simulate a scenario where the query dataset contains unseen cell types, we remove cells belonging to one shared cell type between all reference and query datasets in each test. Each shared cell type is removed once, resulting in multiple tests. For example, with three reference datasets and three cell types shared by all datasets, there will be three tests when using the reference datasets to annotate the query dataset. In the first test, all cells belonging to the yellow cell type in all reference datasets are removed, making the real unseen cell type the yellow cell type. Similarly, in the second and third tests, all cells belonging to the blue and red cell types are removed, respectively. Multiple tests are conducted, with 50 tests for the PBMC collection and 25 tests for the Pancreas collection, by alternatively removing each shared cell type.
(EPS)

S3 Fig Performances in unseen cell-type identification. Boxplots of the AUPRC scores of different methods in (A) PBMC collection and (B) Pancreas collection. The results with different query datasets are displayed in different panels.
(EPS)

S4 Fig Performance summary of mtANN and other compared methods in unseen cell-type identification and cell-type annotation. Bar plots of the number of times each method ranks first in each evaluation metric are illustrated. The evaluation metrics are indicated at the top of the graph and dataset collections are illustrated below the graph. Under each evaluation metric, the top 3 methods are marked with rankings.
(EPS)

S5 Fig Distributions of metrics measuring cell prediction uncertainty when the query dataset is “10X v3” and the real unseen cell type is “B cell”. The distributions of the metric obtained from (A) ItClust, (B) scGCN (enrichment), (C) scGCN (entropy), and (D) scANVI are shown. The color of the histogram distinguishes between the unseen cell type and shared cell types.
(EPS)

S6 Fig Distributions of metrics measuring cell prediction uncertainty when the query dataset is “10X v3” and “CD14+ monocyte” is the real unseen cell type. The distribution of metric obtained from (A) mtANN, (B) scmap-clust, (C) scmap-cell, (D) Seurat v3, (E) ItClust, (F) scGCN (enrichment), (G) scGCN (entropy), and (H) scANVI are illustrated. The color of the histogram distinguishes unseen cell types from shared cell types. The black dotted line in (A) represents the subpopulations of the Gaussian mixture model fitted by mtANN. The grey solid lines in (A-D) represent the default thresholds selected by mtANN, scmap-clust, scmap-cell, and Seurat v3.
(EPS)

S7 Fig Distributions of metrics measuring cell prediction uncertainty when the query dataset is “10X v3” and “Megakaryocyte” is the real unseen cell type. The distribution of metric obtained from (A) mtANN, (B) scmap-clust, (C) scmap-cell, (D) Seurat v3, (E) ItClust, (F) scGCN (enrichment), (G) scGCN (entropy), and (H) scANVI are illustrated. The color of the histogram distinguishes unseen cell types from shared cell types. The black dotted line in (A) represents the subpopulations of the Gaussian mixture model fitted by mtANN. The grey solid lines in (A-D) represent the default thresholds selected by mtANN, scmap-clust, scmap-cell, and Seurat v3.
(EPS)

S8 Fig Performance in unseen cell-type identification under the default threshold. Boxplots of the F1 scores of different methods in (A) PBMC collection and (B) Pancreas collection. The results with different query datasets are displayed in different panels.
(EPS)

S9 Fig Comparison between the true proportion of unseen cell types and the proportion of unassigned cells predicted by each method. Dot plots are displayed for all tests (75 tests) conducted on the PBMC and Pancreas collections, respectively. For each plot, the *x*-axis represents the true proportion, and the *y*-axis represents the proportion of unassigned cells predicted by each method. Each method is denoted with a different color in the plot. The black solid line represents the line of *y* = *x*. Pearson correlation coefficients between the true proportion and the proportion of unassigned cells predicted by each method are reported.
(EPS)

S10 Fig Cell-type annotation performance with the real proportion of unseen cell types as a threshold. Boxplots of the accuracy of different methods in (A) PBMC collection and (B) Pancreas collection. The results with different query datasets are displayed in different panels.
(EPS)

S11 Fig Heatmaps of Pearson correlations between cell-type proportions of the true cell-type label and annotation labels predicted by each method. (A) PBMC collection and (B) Pancreas collection. The columns of the heatmap represent the 50 tests in PBMC collection and the 25 tests in Pancreas collection.
(EPS)

S12 Fig Comparison of cell-type annotation performance between the directly combined references and the corrected references. Boxplots of the accuracy of different methods in (A) PBMC collection and (B) Pancreas collection. The results with different query datasets are displayed in different panels.
(EPS)

S13 Fig Cell-type annotation performance with the default threshold. Boxplots of the accuracy of different methods in (A) PBMC collection and (B) Pancreas collection. The results with different query datasets are displayed in different panels.
(EPS)

S14 Fig Comparison of mtANN’s performance using real proportions versus default threshold for annotation. Boxplots comparing the accuracy of mtANN using the real proportion of unseen cell types versus using the default threshold, for (A) PBMC collection and (B) Pancreas collection. Each panel shows results for a different query dataset.
(EPS)

S15 Fig Comparison between single metrics for unseen cell-type identification and cell-type annotation. Dot plots of all tests (75 tests) conducted in the PBMC and Pancreas collections, respectively. The *x*-axis shows the AUPRC (for unseen cell-type identification) or accuracy (for cell-type identification) of a single metric, while the *y*-axis represents the AUPRC (for unseen cell-type identification) and accuracy (for cell-type identification) of another single metric. Each dot represents an experiment, with different colors representing different data collections. The black solid line corresponds to the line of *y* = *x*. The AR index is reported.
(EPS)

S16 Fig Comparison of runtime and memory usage of all methods. Bar plots comparing the (A) runtimes and (B) memory usage of comparison methods and our method on the PBMC and Pancreas collections. The query dataset for the PBMC collection is “10X v2” and “Baron” is used as the query dataset for the Pancreas collection. All methods are run on a workstation equipped with an Intel(R) Xeon(R) Silver 4214 CPU (2.20GHz x 48), 128GB RAM, and a Tesla V100 PCIe 16GB GPU.
(EPS)

S1 Text Supplementary notes of mtANN. There are algorithm, details in the Modules I-IV of mtANN, methods for benchmark and performance assessment.
(PDF)

S1 Table The query datasets, references and the real unseen cell type of each experiment test in PBMC collection. (DOCX)

S2 Table The query datasets, references and the real unseen cell type of each experiment test in Pancreas collection. (DOCX)

S3 Table Terms and notations. (DOCX)

S4 Table Gene selection threshold settings. (DOCX)

S5 Table The cell types and cell numbers of each dataset in PBMC collection. (DOCX)

S6 Table The cell types and cell numbers of each dataset in Pancreas collection. (DOCX)
