# Supplementary material for: Cell-type annotation with accurate unseen cell-type identification using multiple references
Source: PLoS Comput Biol. 2023 Jun 28;19(6):e1011261. doi: 10.1371/journal.pcbi.1011261 (PMC10335708; doi:10.1371/journal.pcbi.1011261)
Supplement: S1 Table — (DOCX) [file pcbi.1011261.s018.docx]

Table S1 The query datasets, references and the real unseen cell type of each experiment test in PBMC collection
